# Finite element-based evaluation of the supraspinatus tendon biomechanical environment necessitates better clinical management based on tear location and thickness

**DOI:** 10.1038/s41598-024-75339-8

**Published:** 2024-11-01

**Authors:** Mason Garcia, Ahmad Hedayatzadeh Razavi, Daniela Caro, Arun J. Ramappa, Joseph P. DeAngelis, Ara Nazarian

**Affiliations:** 1 Musculoskeletal Translational Innovation Initiative, Beth Israel Deaconess Medical Center, Harvard Medical School, 330 Brookline Avenue, RN123, Boston, MA 02115 USA; 2 Mechanical Engineering Department, Boston University, Boston, MA USA; 3 Carl J. Shapiro Department of Orthopaedic Surgery, Beth Israel Deaconess Medical Center, Harvard Medical School, 330 Brookline Avenue, RN123, Boston, MA 02115 USA; 4 Department of Orthopaedic Surgery, Yerevan State Medical University, Yerevan, Armenia

**Keywords:** Biomedical engineering, Computational science, Computational biophysics

## Abstract

Partial-thickness rotator cuff tears are a common cause of pain and disability and are central to developing full-thickness rotator cuff tears. However, limited knowledge exists regarding the alterations to the mechanical environment due to these lesions. Computational models that study the alterations to the mechanical environment of the supraspinatus tendon can help advance clinical management to avoid tear progression and provide a basis for surgical intervention. In this study, we use three-dimensional validated finite element models from six intact specimens to study the effects of low- and high-grade tears originating on the articular and bursal surfaces of the supraspinatus tendon. Bursal-sided tears generally had a lower failure load, modulus, and strain than articular-sided tears. Thus, caution should be taken when managing bursal-sided tears as they may be more susceptible to tear progression.

## Introduction

Partial-thickness rotator cuff tears (PT-RCT) are common and are an important part of the pathology and natural progression to full-thickness tears^1,2^. Importantly, patients with PT-RCTs experience pain and loss of function, ultimately warranting clinical intervention^2,3^. The incidence of these lesions ranges from 13 to 25% in the general population^4–7^. The cause of PT-RCT tears is multifactorial, with intrinsic degeneration, repeated trauma (overuse), and hypo-vascularity playing roles in developing RC tears^8–10^. Additionally, the supraspinatus plays an important role in the dynamic stabilization of the glenohumeral joint and is the main muscle-tendon unit during abduction^11–15^. Subsequently, it is the most vulnerable structure and is commonly affected by injury and degeneration^2,16–19^.

PT-RCTs, can be classified by location, originating on the articular or bursal surfaces of the supraspinatus tendon^2^. Furthermore, the tendon’s thickness can also be used to classify PT-RCTs. The Ellman classification system groups tears as grade 1 (< 3 mm of tendon thickness), grade 2 (3–6 mm of tendon thickness), and grade 3 (> 6 mm of tendon thickness)^1^. Tears comprising less than 50% of the thickness can be classified as low-grade, and those greater than 50% can be classified as high-grade. The clinical intervention for PT-RCTs often starts with conservative management. However, this approach does not address the underlying tear^20^. As a result, 40% of patients experience tear progression from partial to full-thickness tears within three years^21^.

Previously, Mazzocca et al.. reported that PT-RCTs affecting greater than 50% of the tendon thickness experience significantly higher strain than intact specimens^22^. Due to the consistent use of the shoulder during daily living, the supraspinatus tendon is under constant load, resulting in mechanical damage that may occur over time, causing tears to progress. Alterations to the mechanical environment of the supraspinatus that significantly increase strain may be more likely to experience tear progression. Thus, high-grade PT-RCTs may be better candidates for rotator cuff repair due to changes in the mechanical environment of the supraspinatus tendon.

There is a lack of mechanical data assessing the changes in the mechanical properties of the supraspinatus tendon with PT-RCTs. Furthermore, data assessing the changes in strain of the intact fibers could help guide clinical management. Therefore, we aim to evaluate the mechanical properties of the supraspinatus tendon with low- and high-grade PT-RCTs originating on the articular and bursal surfaces. *We hypothesize that bursal-sided tears will have the lowest failure load*,* and the remaining tissue modulus will decrease with increasing tear thickness*.

## Methods

### Finite element model development

**Fig. 1:**
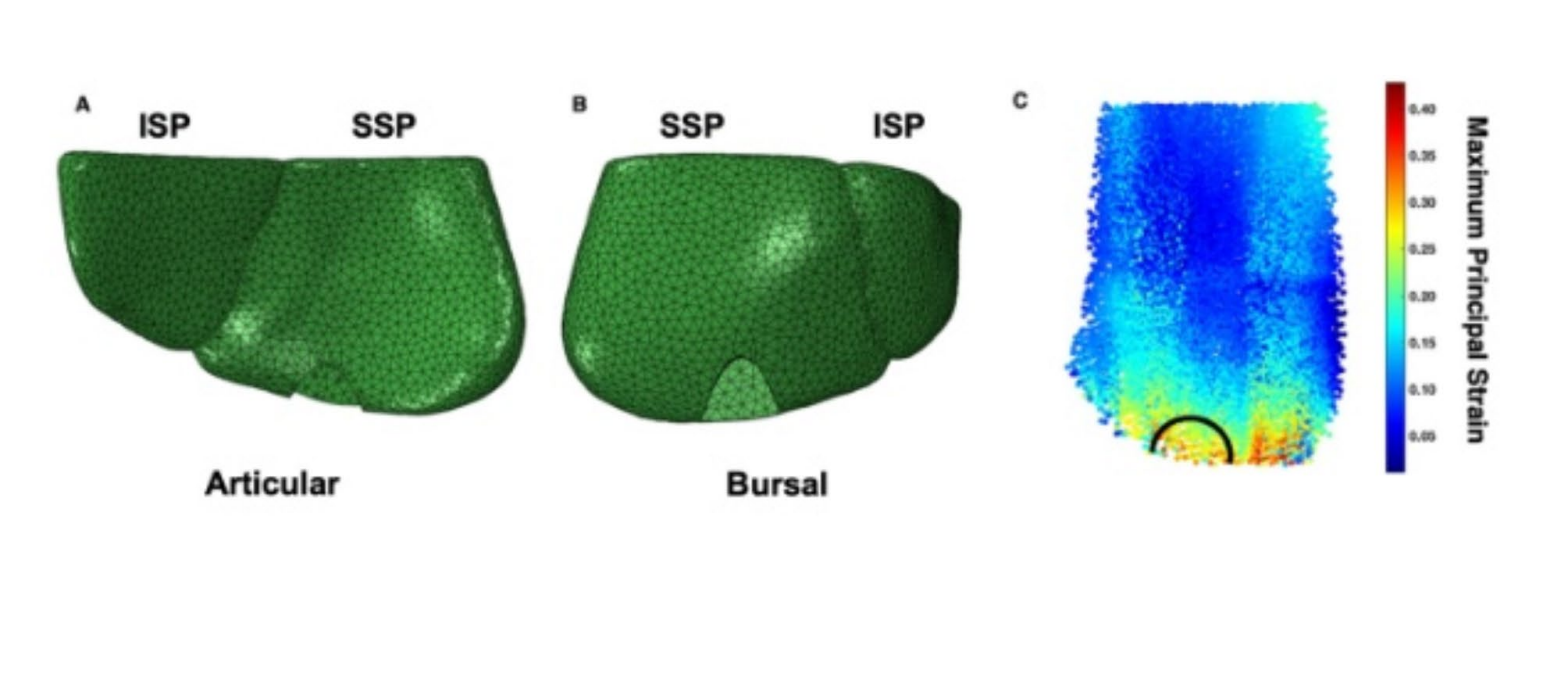
Finite element models of the supraspinatus (SSP) and infraspinatus (ISP) tendons from a left shoulder with articular sided (**A**) and bursal sided (**B**) partial thickness rotator cuff tears. The region of interest where the strain was analyzed for the intact fibers is shown (**C**) for a representative heat map showing the strain when failure strain was reached (images generated using ABAQUS 2023 software package).

The finite element (FE) model was created using a previously validated model of the supraspinatus and the infraspinatus tendons from a left cadaveric shoulder^23^. The model was first validated by applying varying loads to the infraspinatus, assessing the changes in strain due to infraspinatus tendon loads, and comparing the FE-derived surface strain (maximum principal strain) to cadaveric experiments under the same loading conditions^23^. The model geometry of these two tightly connected tendons could predict tissue strain within 3% absolute strain (compared to cadaveric experiments) for infraspinatus loads ranging from 5 to 25 lbs, highlighting the load-sharing interaction of the tendons. To create patient-specific finite element models, six human supraspinatus tendons (mean age: 65 ± 9) were used to determine the heterogeneous material properties^24^. In short, the mechanical response of the supraspinatus tendon was evaluated via uniaxial tensile testing along the medial-lateral axis in 12 distinct regions. These regions allowed the testing of articular/bursal, medial/lateral, and anterior/middle/posterior regions to capture the full mechanical properties. Please refer to Fig. 1 from our previous study^25^ for a schematic representation of the material regions.

Materials were characterized by fitting the uniaxial mechanical data to the Holzapfel – Gasser – Ogden material model, commonly used to model soft tissues^26^. The MATLAB ‘*fminsearch*’ function, where the matrix constant (‘C10’) was set to a constant of 1.0 MPa^27^, and ‘$\:\kappa\:$’ was obtained histologically^25^, the material parameters for the collagen fibers ‘k1’ and ‘k2’ were determined. An example of the spatially varying material properties for each of the 12 regions from one model can be seen in Table 1. Specimen-specific material properties were then applied to the 12 regions of the previously validated model geometry. Each of the 12 regions was created using a custom MATLAB (2023a, MathWorks, Natick, MA, USA) script, which allowed us to split the supraspinatus tendon into 12 equal volumes, matching the 12 regions used for mechanical testing^25^. To further validate the models with varying material properties, the supraspinatus was loaded at 135 N and compared to cadaveric data^28^. Models were considered validated if the maximum principal strain was within 3% of the reported cadaveric strain, as 3% had previously been used as the threshold for model validation^23,29^. All six models were within 3% absolute strain of the experimental data, validating the accuracy of the material properties. Please refer to our previous works for a more comprehensive description of FE model formulation, material testing, and validation^23,25^.

### Finite element model tear creation

Recent studies have suggested that rotator cuff tears originate more posteriorly than previously thought, beginning in the center or posterior half of the supraspinatus tendon^30,31^. Thus, partial-thickness tears were created in the posterior region of the supraspinatus tendon, approximately 16 mm from the anterior edge where the biceps tendon lies, as described *by Kim et al.*^31^. The model was imported into 3-Matic (Mimics Materialize and 3-Matic, Leuven, Belgium), and crescent-shaped tears, as observed clinically, were created on the articular and bursal surfaces of the supraspinatus tendon (Fig. 1A and B). Low-grade tears comprised a tear depth of 33% (1.3 mm) of the tendon thickness, and high-grade tears comprised 66% (2.6 mm) of the tendon thickness^32^. The anterior-posterior (AP) and medial-lateral (ML) dimensions were chosen to represent a PT-RCT seen clinically. Thus, the AP dimension was 6.3 mm, and the ML dimension was 5.4 mm, based on the average tear size from various studies reporting on PT-RCTs^21,33,34^.

### Finite element simulations

FE simulations were conducted in ABAQUS (2023, Dassault Systèmes, Vélizy-Villacoublay, France) using a static simulation over a maximum of 100 steps to ensure convergence of the simulation. The supraspinatus tendon was constrained in all directions from the lateral edge to 1.7 mm above the edge to mimic the supraspinatus osteotendinous insertion^35^ and followed along the infraspinatus. The forces were applied to the medial edge of the supraspinatus and infraspinatus tendons for all models^23^. To determine the failure load, the supraspinatus tendon was loaded until a failure strain of 26.1% was reached in the remaining tendon adjacent to the tear (Fig. 1C)^36^. This failure criterion was chosen as it was reported to be the strain adjacent to the tear tips before critical tear progression occurred. To evaluate the remaining intact tendon, the linear modulus was determined via linear regression from the stress-strain curves constructed from simulation until the failure load of the supraspinatus tendon was reached. The strain of the intact tissue was also assessed at loads of 102 N and 200 N to evaluate how these tears affect the mechanical environment during loading conditions, representing abduction^37^ and low-intensity physical therapy, respectively^29^. To account for the load-sharing interaction between the infraspinatus and supraspinatus, a constant 22 N force was applied to the infraspinatus for all FE simulations^38,39^. The output for all simulations resulted in nodal logarithmic strains and Cauchy stresses, which were converted to maximum principal Lagrangian strains and maximum principal Cauchy Stresses for each node of the supraspinatus tendon.

### Statistical analysis

The Shapiro-Wilk test was used to assess the normality of the data. Normal distribution was reported for FE data. Data were analyzed using one-way analysis of variance (ANOVA). The Tukey *post-hoc* test was used for multiple comparisons of simple effects of the tear type on failure load and the multiple comparisons of simple effects of the tear type on intact tissue modulus. Statistical analysis was performed using GraphPad Prism unless otherwise noted (version 9.3.1 for Windows, GraphPad Software, San Diego, CA, USA). Two-tailed p-values less than 0.05 were considered significant.

## Results

### Failure load

**Fig. 2:**
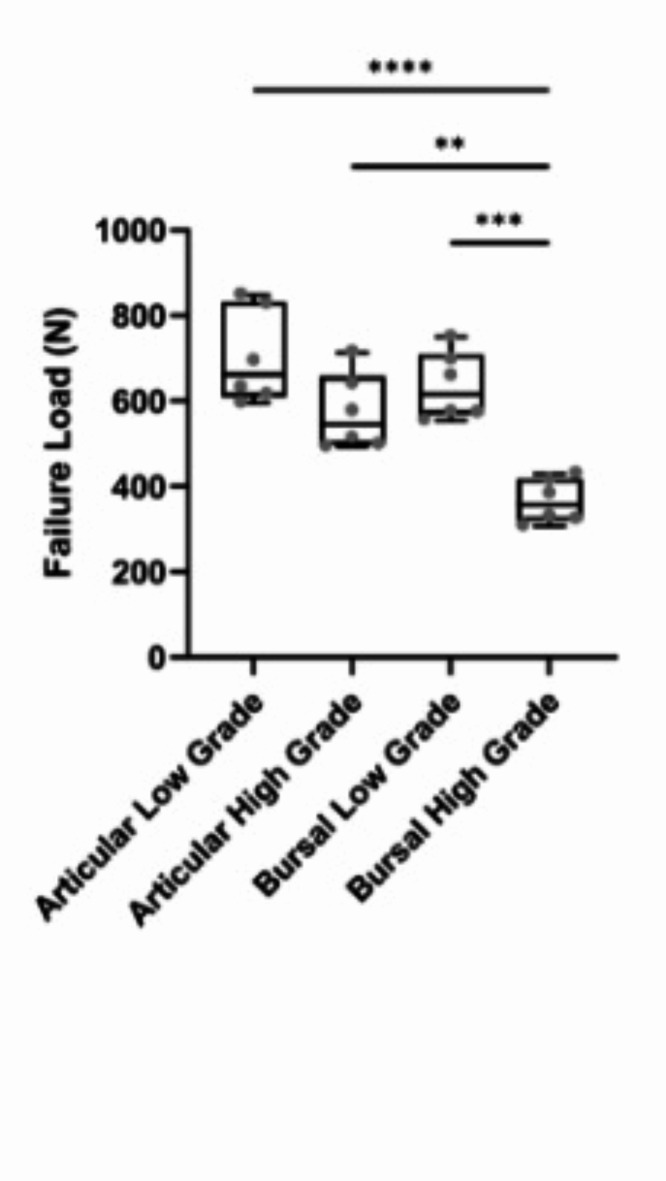
Failure load determined from finite element models for each tear type. ‘*’ represents *p* < 0.05, ‘**’ represents *p* < 0.01, ‘***’ represents *p* < 0.001, and ‘****’ represents *p* < 0.0001.

The failure load for each model was determined to compare the alterations to the mechanical environment of the supraspinatus tendon with increasing tear depth (Fig. 2). Failure load for articular low-grade tears was 701.6 ± 110.4 N (range: 595.5–847.5 N), with a 34.9% maximum percent difference between specimens. Failure load for articular high-grade tears was 572.2 ± 89.3 N (range: 493.1–713.4 N), with a 36.5% maximum percent difference between specimens. The failure load for bursal low-grade tears was 643.3 ± 79.2 N (range: 554.8–749.5 N), with the maximum percent difference between specimens being 29.9%. The failure load for bursal high-grade tears was 364.7 ± 51.3 N (range: 307.2–429.9 N), with the maximum percent difference between specimens being 33.3%. In general, failure load decreased with increasing tear depth for both articular- and bursal-sided tears. However, no significant difference was observed between low- and high-grade articular PT-RCTs. Bursal-sided low-grade PT-RCTs demonstrated significantly larger failure loads (*p* = 0.001). Articular-sided PT-RCTs had higher failure loads than bursal-sided tears. Low-grade articular-sided tears had a significantly higher failure load than high-grade bursal tears (*p* < 0.0001). Furthermore, articular high-grade tears had a significantly higher failure load than bursal high-grade tears (*p* = 0.002).

### Modulus of intact tissue

**Fig. 3:**
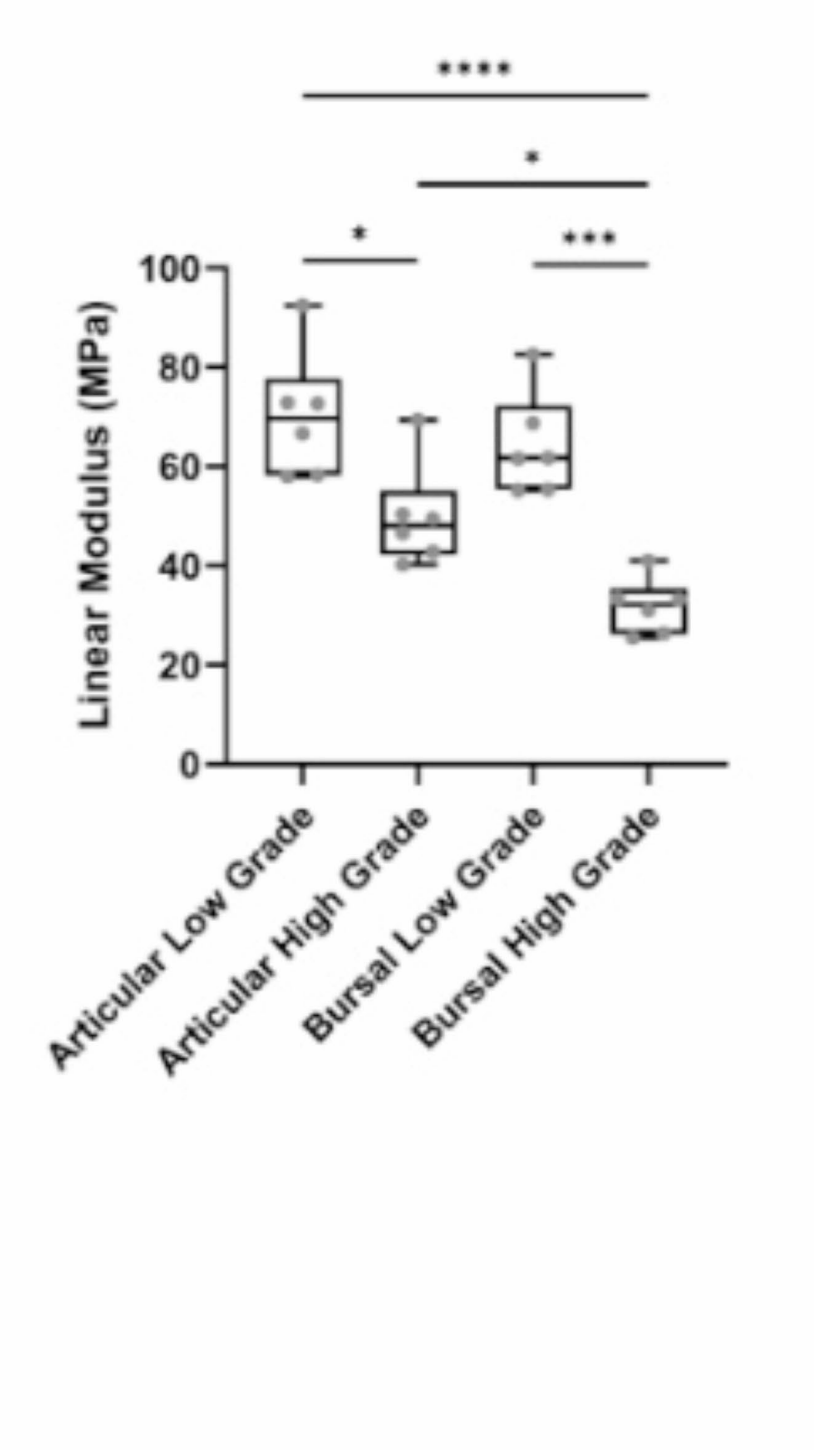
Linear modulus of intact tissue determined from finite element models for each tear type. ‘*’ represents *p* < 0.05, ‘**’ represents *p* < 0.01, ‘***’ represents *p* < 0.001, and ‘****’ represents *p* < 0.0001.

The intact tissue behind the tear was analyzed to determine its linear modulus and to evaluate mechanical behavior (Fig. 3). The linear moduli of the intact tissue were 70.2 ± 12.7 MPa for low-grade articular-sided tears and 49.8 ± 10.3 MPa for high-grade articular-sided tears. Similarly, the linear moduli of the intact tissue were 64.2 ± 10.3 MPa for low-grade bursal-sided tears and 31.8 ± 5.6 MPa for high-grade bursal-sided tears. Similar to what was seen for the failure loads, the moduli of the remaining tissue behind the tear site decreased with increasing tear depth, with articular-sided tears having a higher linear modulus than bursal-sided tears. The linear modulus of low-grade articular-sided tears was significantly higher than high-grade articular-sided tears (*p* = 0.013). Furthermore, the linear modulus of low-grade bursal-sided tears was significantly higher than high-grade bursal-sided tears (*p* < 0.0001). While no significant differences were observed when comparing articular and bursal low-grade tears, articular low- and high-grade tears had a significantly higher linear modulus than bursal high-grade tears (*p* < 0.0001 and *p* = 0.027, respectively).

### Strain during abduction and physical therapy

**Fig. 4:**
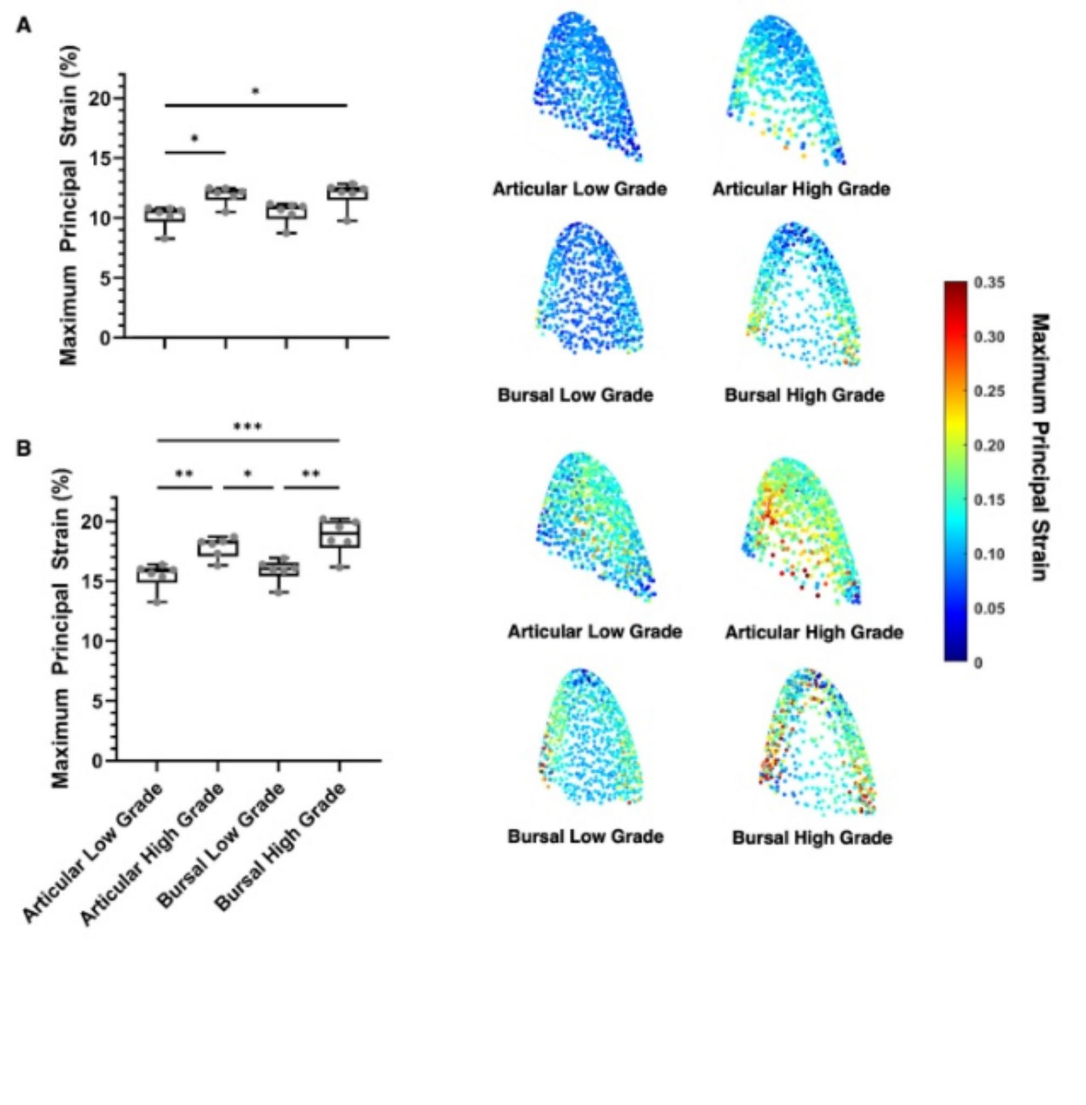
Average maximum principal strain within the region of interest (intact tissue behind tear) for (**A**) simulated abduction and (**B**) low intensity physical therapy. Heat maps show distribution of strain in the remaining intact fibers that was averaged together to assess the mechanical properties of the tendon.

During loading conditions that simulated abduction (102 N), strain increased with tear size; however, the increase was very small (Fig. 4A). The average maximum principal strains of the intact tissue for low- and high-grade articular-sided tears were 10.0 ± 1.0% and 11.9 ± 0.7%, respectively. The average maximum principal strains of the intact tissue for low- and high-grade bursal-sided tears were 10.5 ± 0.9% and 12.0 ± 1.1%, respectively. Low-grade articular-sided tears had significantly less strain than high-grade articular-sided (*p =* 0.021) and bursal-sided (*p =* 0.018) tears.

During loading conditions that simulated physical therapy (200 N), the strain increase with tear size was more pronounced (Fig. 4B). The average maximum principal strains of the intact tissue for low- and high-grade articular-sided tears were 15.4 ± 1.1% and 17.9 ± 0.9% strain, respectively. The average maximum principal strains of the intact tissue for low- and high-grade bursal-sided tears were 15.9 ± 1.0% and 18.8 ± 1.5% strain, respectively. Low-grade articular-sided tears experienced the lowest strain, having significantly less strain than high-grade articular-sided (*p* = 0.008) and bursal-sided (*p* < 0.0001) tears. High-grade bursal-sided tears had the highest strain, having significantly more strain than low-grade bursal-sided tears (*p* = 0.001).

## Discussion

This study describes the mechanical environment of the supraspinatus tendon with varying degrees of PT-RCTs. Evaluation of the failure load, tissue modulus, and intra-tendinous strain helps to explain why some tears are more likely to progress than others. Furthermore, a better understanding of the mechanical environment can help guide clinical management based on the altered mechanical properties of the supraspinatus tendon. Our study revealed that regardless of location (articular versus bursal), low-grade tears experience significantly higher failure loads, intact tissue modulus, and significantly less strain during physical therapy. High-grade articular-sided tears saw a 22.6% decrease in failure load and a 40.8% decrease in intact tissue modulus compared to low-grade articular-sided tears. Furthermore, high-grade bursal-sided tears saw a 42.5% decrease in failure load and a 50.5% decrease in intact tissue modulus compared to low-grade bursal-sided tears. Additionally, we observed that bursal-sided tears had significantly lower failure loads and experienced more strain than articular-sided tears, suggesting that bursal-sided tears may be more likely to progress. These results support our hypothesis that bursal-sided tears have decreased mechanical function, showing significantly lower failure loads compared to articular-sided tears.

Previous studies that have evaluated the mechanical environment of an intact RC are important to consider when evaluating the effects PT-RCTs have on the supraspinatus tendon. Previous studies have shown that under uniaxial loading conditions, similar to what is seen here, the insertion is under significantly higher strain than the mid-tendon and the musculotendinous junction^28^. However, this study did not note any differences in strain in anterior, middle, or posterior regions, suggesting that the strain along the tendon in the anterior-posterior region is homogenous under uniaxial loading. The strain distribution changes in the presence of a full-thickness RC tear, resulting in stress concentrations at the tear tips^25,36^. Like the presence of a full-thickness tear, the loss of tissue results in an altered strain pattern, where higher strains are seen in the remaining intact tissue and around the tear tips. This alteration is important because repetitive trauma to a singular region or overload could further damage this area, resulting in tear progression.

The mechanical properties of the supraspinatus tendon are altered as a function of tear depth, showing increases in strain and, with every 25% increase in tear size, an 182 N decrease in failure load^22^. One result of this increased strain in the remaining tissue could explain the apparent decrease in tissue modulus. This could be due to the strain concentration that arises in the torn area, as at this localized region, we see higher strain values giving rise to an area subject to damage and degeneration and further alterations to the mechanical function. The previously reported failure load for low-grade articular-sided PT-RCTs was approximately 600 N; our study found low-grade articular-sided tears had a failure load of 701 N. For high-grade articular-sided PT-R – RCTs, the cadaveric failure load was reported to be 236 N, and our study found that high-grade articular-sided PT-RCTs had a failure load of 572 N. The difference in observed failure loads could be due to the differences in tear size. The cadaveric study^22^ created tears that spanned 1/3 of the supraspinatus tendon footprint, representing an 8.33 mm wide tear based on the average footprint width^35^. Furthermore, the cadaveric low-grade tears had a tear depth of 25% of the thickness, whereas our models had a tear depth of 33%. The high-grade tears had a tear depth of 75%, whereas the model had a tear depth of 66%. Nonetheless, we show similar failure loads and the same trends of decreasing mechanical function with increasing tear depth.

The optimal management of PT-RCTs remains unclear. The American Academy of Orthopedic Surgeons (AAOS) has developed clinical guidelines for treating rotator cuff tears^40^. However, limited data has led to the inability to endorse debridement versus repair in high-grade tears, trans-tendon versus conversion to full-thickness repairs, or management of low-grade tears. Furthermore, no guidelines indicate different managements for articular-sided versus bursal-sided tears. Typically, surgical intervention is reserved for patients who initially fail conservative management^40^. However, based on the changes to the mechanical environment of the supraspinatus tendon due to different tear types, some patients may not be best suited as it could result in larger and more complex tears.

While PT-RCTs are less likely to progress than full-thickness tears and have a lower risk of fatty infiltration and muscle atrophy, there remains limited evidence supporting non-operative management^41–43^. Furthermore, when surgery should be considered remains largely unknown. One study has examined changes in strain due to tear depth, finding that strain significantly increases once tears affect greater than 50% of the tendon thickness; however, they only studied articular-sided tears^22^. Furthermore, one study examining the outcomes of physiotherapy on PT-RCTs found that patients with low-grade PT-RCTs (< 50% thickness) were more likely to be relieved of symptoms^20^. In support of these findings, both articular-sided and bursal-sided tears saw significant decreases in failure loads (22.6% and 42.5%, respectively) and moduli (40.8% and 50.5%, respectively). Although our findings agree with this approach, it may be important to consider the differences in the biomechanical environment between articular- and bursal-sided tears, as bursal-sided tears are at higher risk for progress. Due to the significant differences in the mechanical environment of high-grade bursal-sided tears versus articular-sided tears, it could be suggested that articular-sided tears respond more favorably to non-operative management.

There is limited knowledge regarding the optimal treatment for articular- and bursal-sided PT-RCTs, as studies typically group tears together or only focus on one tear type. However, a recent review on PT-RCT management has shown that bursal-sided tears respond less favorably than articular-sided tears after arthroscopic debridement^44^. Arthroscopic debridement does not repair the tears. Instead, a shaver is used to remove frayed edges around the tear^43^. Since no biomechanical augmentation (i.e., surgical fixation) is performed, the remaining tissue bears all the load. Our study has confirmed that bursal-sided tears have decreased biomechanical properties compared to articular-sided tears.

The optimal technique for surgery remains debated, with trans-tendon and conversion to full thickness being the two most common approaches^45,46^. In support of trans-tendon repair, the remaining tissue is left preserved, which could help increase the strength of the repair^47,48^. When comparing trans-tendon repair versus conversion to full thickness, a recent systematic review and meta-analysis showed no significant differences in structural, functional outcomes, or re-tear rates^44^. However, it should be noted that these studies largely focused on high-grade tears. Interestingly, despite our model’s significant decrease in mechanical properties (failure load and modulus), clinical findings show that trans-tendon repair and conversion to full thickness offer equivalent functional and structural outcomes^44^. This finding suggests that while significant decreases in the mechanical properties of the rotator cuff are seen, they are not enough to worsen outcomes in one method versus another. These studies showed that re-tear rates were higher for bursal-sided tears when converted to full-thickness tears, possibly due to the significant disruption to the mechanical environment (decreased failure load and modulus) in bursal-sided tears compared to articular-sided tears.

Some authors have recommended a differential approach to PT-RCT repairs based on the tendon thickness affected. *Park et al.* suggested trans-tendon repair when roughly 50% of the thickness is affected and conversion to full-thickness when 90% is affected^49^, with others following a similar approach^44^. While many studies focus on the repair of high-grade tears, some studies have reported outcomes of low-grade tears^50,51^. However, there has not been a comparison between trans-tendon repair and conversion to full thickness for low-grade tears. Since there are no differences in clinical or structural outcomes between these two techniques even after significant decreases in the mechanical environment, one may consider the possibility of keeping the intact tendon when repairing low-grade PT-RCTs due to enhanced mechanical properties of the tendon compared to high-grade PT-RCTs. However, clinical studies are needed to confirm this hypothesis.

Many factors should be considered when managing PT-RCTs, such as location (bursal versus articular) and tear depth (high vs. low grade)^46,52,53^. Because mechanical data describing the alterations to the mechanical environment of the supraspinatus tendon due to PT-RCTs are scarce, our study evaluated the mechanical properties, such as failure load, intact tissue modulus, and tendon strain, to better inform the clinical management of PT-RCTs. Our study found that bursal-sided tears are significantly weaker than articular-sided tears, and they may respond less favorably to non-operative interventions such as physical therapy due to an increased risk of tear progression. Due to this concern, patients with bursal-sided tears may benefit from early surgical intervention. Furthermore, we found a significant decrease in mechanical properties in high-grade tears, suggesting that high-grade tears will be more prone to progress than low-grade tears. It has been shown that there are no clinical differences in terms of functional and structural outcomes for trans-tendon and conversion to full-thickness repairs for high-grade tears^44^. However, low-grade tears that fail non-operative management may benefit from preservation of the intact fibers of the tendon, as their superior mechanical properties may increase the tensile strength of the repair. Future research should focus on comparing articular- and bursal-sided tears to understand better how different management techniques can provide more optimal treatment methods.

## Limitations

Several limitations should be addressed in this study. The first is using cadaveric tissue to simulate the in-vivo tendon strain. However, this limitation is shared among all cadaveric and simulation studies evaluating RC mechanics^54^. Furthermore, this model only includes the supraspinatus and infraspinatus tendon, not the other RC muscles. This can be explained by the fact that the teres minor and subscapularis do not insert with the supraspinatus as the infraspinatus. The teres minor and subscapularis were excluded because they have minimal loading effect on the supraspinatus tendon^55^. Furthermore, the infraspinatus and the supraspinatus are tightly connected and have a load-sharing interaction where increased loads on the infraspinatus alter the supraspinatus tendon strain^32,55,56^. While this study did not account for higher infraspinatus loads that may be seen in-vivo, which could potentially decrease the supraspinatus tendons failure due to the load-sharing interaction, we focused on the load-bearing capacity and mechanical function of the supraspinatus tendon only, which can be better compared to previous cadaveric experiments. Another limitation is the use of static loading conditions. While the RC is dynamic in nature and undergoes many cycles of loading, this is a common limitation with all biomechanical studies of the RC. Moreover, understanding the failure loads makes an important distinction into which constructs are mechanically stronger. Lastly, our model did not include the bone geometry of the humeral head, which is due to the scanning protocol used to generate the initial model geometry, which was tuned for soft tissue^23^. However, since the bone has a significantly higher modulus than the tendon (GPa vs. MPa), it is unlikely any deformation will occur to the bone. This distinction between material properties is simplified to a rigid boundary condition, resulting in the highest strain occurring at the insertion, commonly seen in cadaveric studies^28^. While our model and previous cadaveric studies point to decreased mechanical function with increasing tear depth, biomechanical studies are needed to elucidate these results further using cadaveric specimens with pre-existing RC tears to evaluate if the current cadaveric RC tear models and FE models behave similarly.

## Conclusion

We have developed finite element models of PT-RCTs with varying severity to identify alterations to the mechanical environment of the supraspinatus. This study gleans insight into the alterations to the mechanical environment of the supraspinatus tendon with varying tear chronicity. This allows us to understand better how tear depth changes in PT-RCTs affect tendon response to load and the differing mechanical properties between articular and bursal-sided tears. We have shown that bursal-sided PT-RTCs are mechanically weaker and may be more likely to experience tear progression. These tears should be treated more cautiously when deciding surgical vs. non-operative intervention to limit the risk of tear progression. While this study provides insight into the varying mechanical properties of PT-RCTs, clinical studies are needed to confirm these findings and identify the optimal treatment methods.

**Table 1:** Specimen specific material parameters obtained and used for one specimen.

Region	k1 (MPa)	k2	κ
ALA	1.96	31.32	0.089
ALM	0.99	32.62	0.055
ALP	0.94	19.45	0.037
AMA	0.77	32.24	0.024
AMM	0.57	38.18	0.055
AMP	0.78	21.91	0.058
BLA	1.56	27.93	0.079
BLM	0.97	39.49	0.079
BLP	0.59	56.06	0.102
BMA	1.00	50.75	0.091
BMM	0.63	42.10	0.080
BMP	1.76	26.46	0.096

## Data Availability

The datasets used and/or analyzed during the current study are available from the corresponding author upon reasonable request.
